# Using Equivalence-Based Instruction to Teach Value-Congruent Action Identification to Autistic Teens

**DOI:** 10.1007/s40617-025-01152-y

**Published:** 2026-02-23

**Authors:** Amanda N. Chastain, Mark R. Dixon

**Affiliations:** 1 Department of Disability and Human Development, College of Applied Health Sciences, Chicago, IL 60608 USA; 2 Oregon Institute of Technology, Klamath Falls, OR USA

**Keywords:** Derived relational responding, Autism, Derived intraverbal, ACT, Values

## Abstract

The current clinical evaluation used a multiple baseline across participants design to assess the effectiveness of intraverbal training in promoting value-congruent action identification among three autistic teenagers. Participants were taught to verbally relate value-congruent actions (A) to outcomes (B), and outcomes (B) to participant-identified values (C), with tests for emergent C-A relations. Two participants demonstrated emergent value-to-action intraverbals without direct training, while one required exemplar training for partial emergence. These findings support the use of relational training to establish derived intraverbals linking values to verbal identification of value-congruent actions. The approach may be especially useful in neurodiversity-affirming clinical practice to support clients in identifying their own value-congruent actions.

Emerging from the broader milieu of the neurodiversity movement, behavior analysts who align with its principles are calling for a more compassionate, client-centered approach; one that affirms autonomy and places the values of neurodivergent individuals at the center of their own programming (Rodriguez et al., 2024; Werntz et al., 2023). Empirical demonstrations specifically aimed at aligning existing behavior analytic technologies with values of dignity, autonomy, and person-centered care may support this evolving practice. One such approach is Acceptance and Commitment Therapy (ACT; Hayes et al., 2006), sometimes referred to as Acceptance and Commitment Training in non-psychotherapeutic applications (ACTr; Tarbox et al., 2021), which offers a framework for individualized, value-congruent behavior change. Rooted in Relational Frame Theory (RFT; Hayes et al., 2001), which is a behavior analytic theory of language and cognition, ACT is described as a value-driven approach that promotes flexibility, increases contact with reinforcement, and enhances quality of life by transforming the function of aversive stimuli into appetitive ones. This transformation is believed to allow individuals to more readily access reinforcing consequences in their present environment (Bordieri, 2022; Dixon et al., 2023; Tarbox et al., 2021).

Central to ACT is clarifying an individual’s values and identifying the value-congruent actions aligned with them. Values can be operationalized as verbal stimuli that alter the reinforcing effectiveness of stimuli (i.e., augmental), either by establishing stimuli as new reinforcers (i.e., formative augmental) or increasing the momentary effectiveness of an already established reinforcer (i.e., motivative augmental) (Tarbox et al., 2021). Put simply, values are verbally constructed reinforcers that serve as establishing operations for engaging in value-congruent behavior. By establishing one’s value as a verbally constructed reinforcer, alternative and value-congruent behavior patterns can be strengthened, ultimately increasing access to more value-congruent reinforcers. For instance, an individual who values kindness may demonstrate this through behaviors such as offering help, expressing empathy, and providing sincere compliments in their daily interactions. Supporting with the identification of values-congruent actions may be of utility to the autistic person who, like many people, may struggle to engage in behaviors aligned with their values on a day-to-day basis.

ACT exercises often involve committing to self-identified value-congruent actions, which involves verbally relating values, actions, and the outcomes of those actions. This might be conceptualized as a stimulus equivalence paradigm (Sidman, 1971), in which a relationship is taught between a spoken action and spoken outcome (A = B) as well as between the spoken outcome and a client value (B = C). Responding accurately to the question, “What action is in line with your value of [client-specific value]?” (C = A) reflects a verbal relation consistent with the derived intraverbal literature base (see Conine et al., 2024 for a review of the literature on derived intraverbals). The present clinical evaluation provides a demonstration of how practitioners might use equivalence-based instruction to establish derived intraverbals between client-stated values and descriptions of value-congruent actions. Through intraverbal training, three autistic teenagers were taught to relate a verbal action (A) to a verbal outcome (B), and the verbal outcome (B) to a client-identified value (C). Tests were then employed to assess the derived intraverbal relations between client-identified value and verbally stated action (C-A) were then conducted to test for value-congruent action identification.

## Method

### Participants, Setting, and Materials

Three teenagers diagnosed with autism spectrum disorder (ASD), and who did not vocally identify value-congruent actions when instructed to state behaviors aligned with their values, participated in this clinical evaluation. Each received weekly 60–90-min ABA sessions focused on social-emotional development using ACT at a small midwestern university clinic. The PEAK Comprehensive Assessment (PCA; Dixon, 2019) was administered to assess each participant’s verbal skills across five modules—direct training, generalization, equivalence, transformation receptive, and transformation expressive—comprising a total of 344 test items, with each successive module reflecting increasing complexity of verbal behavior.

Kenju was a 13-year-old Asian boy who attended weekly 90-min ABA sessions. Kenju’s total PCA score was 302 out of 344 (88%), with notable differences in performance (below 80% overall) on subtests for receptive coordination (75%), receptive opposition (50%), expressive comparison (75%), and expressive hierarchical framing (75%). Diego was a 12-year-old Hispanic boy with a co-occurring diagnosis of attention-deficit/hyperactivity disorder (ADHD) combined type who attended weekly 60-min ABA sessions. His total PCA score was 263 out of 344 possible (77%), with notable differences in performance (below 80% overall) on subtests for equivalence (67%), receptive coordination (50%), receptive comparison (69%), receptive (13%) and expressive (75%) distinction, expressive hierarchy (50%), and receptive (69%) along with expressive (56%) deictic framing. Finally, Ethan was a 15-year-old boy who attended weekly 60-min ABA sessions. His total PCA score was 303 out of the total 344 (88%), with notable differences in performance (below 80% overall) on subtests for transitivity (67%), expressive coordination (63%), and expressive deictic (31%) framing.

Participant values were identified prior to the onset of the current evaluation using activities from the first three modules of the AIM Curriculum (Dixon & Paliliunas, 2018). At the beginning of their regularly scheduled ABA sessions, participants were exposed to one or two AIM activities targeting various ACT-based repertoires, including values identification exercises. Additionally, during the first month of ABA intervention, participants were asked to identify their “three wishes” if they could wish for anything. When responses included tangible or achievable outcomes (e.g., “I wish always be with my cat”), follow-up questions such as “Why?” were used to evoke descriptions of more abstract values (e.g., “I have fun with my cat and I like having fun”). It is important to note that values identification was not the focus of this clinical evaluation, thus a systematic and technological description of this process is not described here. Kenju’s pre-identified values included having fun, getting a good education, and family. Diego’s pre-identified values included family, football, and friends. Finally, Ethan’s pre-identified values were decreasing pain and suffering, understanding, and efficiency. Table 1 depicts verbal stimuli used for each participant.

**Table 1:** Training and testing stimuli

Stimulus class	Action (A stimuli)	Outcome (B stimuli)	Value (C stimuli)
Kenju			
1	Play with Cashmiere	Cashmiere purrs	Having Fun
2	Ask for help if you don’t understand	Get help	Education
3	Give brother a turn	Brother will have fun	Family
Diego			
1	Acting respectfully at school	Getting a gold or platinum day	Family
2	Practicing present moment awareness	Playing a better game	Football
3	Helping a peer with a task	Peer does well with task	Friends
Ethan			
1	Random act of kindness	Make someone smile	Decrease pain and suffering
2	Listen to others	Gain perspective	Understanding
3	Put shoes where they belong	Know where shoes are	Efficiency

### Dependent Variables

The primary dependent variables were percentage of correct intraverbal responses for directly trained (A-B and B-C) relations and tested (C-A) relations made up of three-three members stimulus classes (Table 1). Participants were taught to relate a spoken action (A) to a spoken outcome of that action (B), and to relate the spoken action outcome (B) to a spoken client identified value (C). During A-B training, responses were scored as correct if the participant stated the action outcome belonging to the same stimulus class as indicated on Table 1, or a similar phrase consisting of the core elements of the target response. For example, during A-B probes, Diego might have responded with “Getting a gold or platinum day at school” or “I get a gold or platinum day at school” and both would be noted as a correct response. On B-C test trials, the instructor asked, “What value is (B) aligned with?” to assess for the intraverbal relation between the actions outcome (B) and the participant’s value (C). A trial was scored as correct if the participant stated the value that corresponded with the action-outcome (see Table 1). For example, when given the phrase, “What value is your brother having fun aligned with?” a correct response for Kenju would be the vocal response, “Family”). All other responses were recorded as incorrect. Finally, during the C-A probe and exemplar training conditions, a correct response consisted of the participant stating the action that corresponded with that value as indicated on Table 1. Continuing with the example with Kenju, a correct response to the question, “What could you do to work toward a value of family?” would be to say, “give my brother a turn.”

### Interobserver Agreement and Procedural Fidelity

Interobserver agreement (IOA) was calculated for a minimum of 33% of trials across all conditions using the formula for total agreement [(total trials in agreement/total trials) X 100]. IOA was reported to be above 90% for all training and testing conditions. Procedural fidelity was calculated for a minimum of 33% of trials across all conditions. A trial was scored as correct if the correct instruction was delivered and the correct consequence (i.e., neutral feedback for test trials, praise or an error statement for training trials) was delivered within 2 s of the participants’ response. A trial was scored as incorrect if any of these conditions were not satisfied. The total procedural fidelity was calculated for each block using the formula [(total trials in agreement/total trials) X 100] and was reported to be above 90% for all training and testing conditions.

### Procedure

The experimental protocol, adapted from the PEAK Relational Training System, Equivalence Module (Dixon, 2015), was run once per ABA session as a part of the participant’s regular program goals. Target stimuli consisted of spoken value-congruent actions (A), spoken outcomes of those actions (B), and spoken participant values (C). Spoken actions and outcomes of those actions were created by the experimenter based on known participant values and common activities. A nonconcurrent multiple baseline design across participants was used to evaluate the effectiveness of equivalence-based instruction on the establishment of derived intraverbal relations between participant values (C) and value-congruent actions (A) following direct training on A-B (actions to outcomes) and B-C (outcomes to values) intraverbal relations.

Across all conditions and phases, except for C-A exemplar training, each block consisted of 10 trials, in which each sample stimulus was presented a maximum of four times and for no more than two consecutive trials within each 10-trial block. Targets were presented semi-randomly within a 10-trial block such that each target was presented at least three times, and no target was presented for two consecutive trials. The target that was presented a fourth time was rotated pseudo-randomly throughout the duration of the experiment in an effort to reduce over presentation of any one stimulus set. Training and testing blocks were presented sequentially, with A-B training presented first, followed by B-C training, and C-A testing. Within C-A exemplar training blocks, each sample stimulus was presented three times in a pseudorandomized order, totaling nine trials per block. For all trials, participants were allotted up to 5 s to respond and the first response was recorded as their answer. Program mastery was defined as responding at or above 90% accuracy (9 out of 10 correct trials per block) across two consecutive blocks for each relation type. Exemplar training was introduced for C-A relations if participants failed to meet mastery across three consecutive C-A test blocks, despite meeting criteria for A-B and B-C relations.

#### Baseline

At the onset of the evaluation, baseline data were collected across A-B, B-C and C-A relations, presented sequentially. The number of probe blocks was staggered across participants according to the experimental design. During A-B probes, the instructor asked, “What will happen if you (A)?” to assess the intraverbal relation between a value-congruent action (A) and its outcome (B). On C-A baseline probes, the instructor asked, “What could you do to work toward your value of (C)?” to assess for the derived relation between participant value (C) and value-congruent action (A). During all baseline probes, correct and incorrect responses resulted in neutral feedback (e.g., “okay,” “thank you”) followed by the presentation of the next trial.

#### Relational Training and Testing

After baseline probes, A-B training, B-C training, and C-A testing were presented sequentially until program mastery. During training blocks, correct responses resulted in praise, while incorrect responses resulted in a varied “no” statement (e.g., “that’s not quite right,” “no, let’s try that again”). Contingent on two consecutive errors on a given trial, prompts were introduced. These included priming strategies (i.e., using a verbal prompt prior to delivering the question), partial verbal prompts, and full verbal prompts as needed. While this did not occur, if a participant failed to respond to the prompt during the error correction procedure, the trial would have been re-presented up to two additional times with the error correction before being scored as a nonresponse (i.e., a score of 0). All other procedural parameters mirrored those used during baseline probes for each target relation. Procedural parameters for C-A testing blocks were identical to C-A baseline probes.

#### Exemplar Training

Diego did not meet mastery criterion during the previous phases and required exemplar training. During this condition, C-A exemplars were presented sequentially with A-B and B-C training. Feedback was initially given for participant responses to stimulus class 1, while class 2 and 3 remained in testing. Exemplar training for stimulus class 2 was introduced after the participant completed three consecutive blocks of stimulus class 1 C-A training with 100% accuracy (3 out of 3 trials) without reaching mastery for the entire C-A block. Programmed consequences for trained relations matched those outlined during A-B and B-C training above. Mastery criteria for exemplar training on C-A relations was eight out of nine correct independent responses (89%) on C-A blocks while maintaining above 90% on A-B and B-C training blocks for two consecutive rounds of A-B, B-C, and C-A relations.

## Results and Discussion

**Fig. 1:**
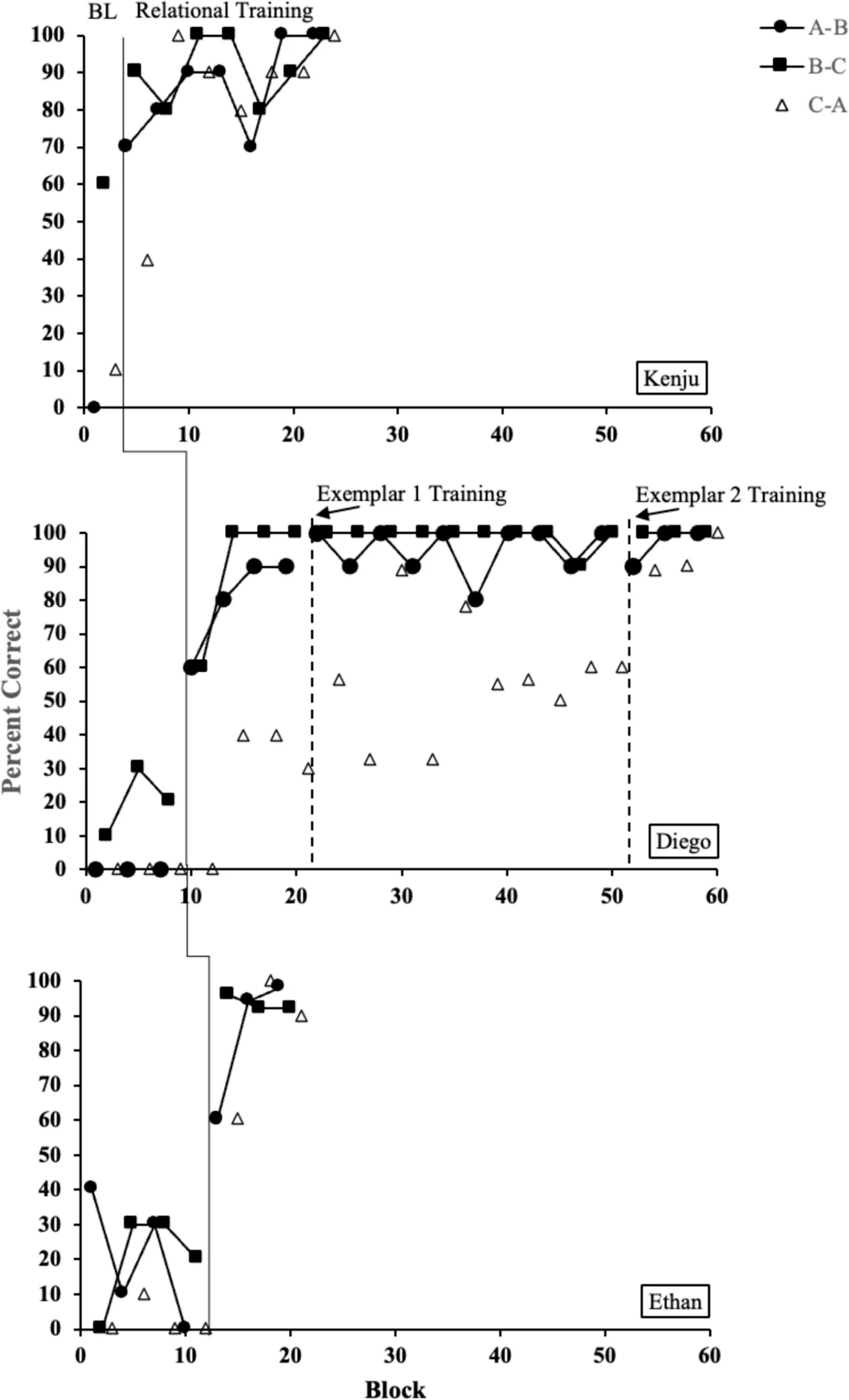
Participant data. *Note.* BL indicates baseline probes. Trained relations are depicted using connected markers while untrained (tested) relations are depicted using disconnected markers. Relational training phase consisted of A-B and B-C relational training, with C-A testing. Diego required exemplar training for class 1 (C1) and class 2 (C2)

Figure 1 depicts the participants’ scores for training (A-B and B-C) and testing (C-A) relations. All three participants performed below mastery criterion for all pretest blocks for A-B (range 0–40%), B-C (range 0–60%), and C-A (range 0–10%). Deigo received three blocks of baseline probes due to an increase in score from 10 to 30% on the first and second B-C probe blocks. The score reduced to 20% on the third attempt. Percentage of correct value-drive action (A) to outcome (B), and outcome (B) to value (C) intraverbals increased for all three participants once training was introduced. Kenju reached mastery after seven blocks of A-B training (range 70–100%), B-C training (range 80–100%), and C-A testing (range 40–100%), with no training required for C-A relations. Similarly, Ethan reached mastery after four blocks of A-B training (range 50–100%), B-C training (range 80–90%), and C-A testing (range 60–100%), with no training required for C-A relations. Diego reached mastery levels of performance for A-B (range 60–100%) and B-C (range 60–100%) training; however, his C-A test performance remained low (range 0–40%), consistently responding with the outcome (e.g., C1-B1) rather than the corresponding action (e.g., C1-A1). As a result, exemplar training was introduced for class 1, with feedback provided only for the C1-A1 relation. Mastery for class 1 was achieved after nine blocks of C-A exemplar training (3/3 correct C1-A1 trials within the larger nine-trial block), though overall C-A accuracy remained variable (range 33–100%). Exemplar training was then extended to class 2, with feedback on C1-A1 and C2-A2 relations, while C3-A3 remained under testing conditions. Mastery across all C-A relations was achieved following this phase (89–100%) without direct training for class 3. Throughout exemplar training, Diego’s performance on A-B and B-C training blocks remained high, ranging from 80–100% and 90–100%, respectively.

The present demonstration assessed the effectiveness of a relational training procedure to teach value-congruent action identification to three autistic teenagers. Participants were directly taught to relate value-congruent actions (A) to outcomes of those actions (B), and outcomes of those actions (B) to participant-identified values (C). Tests were employed to evaluate the emergence of intraverbal relations between participant-identified values (C) and value-congruent actions (A). All three participants met mastery criteria for training conditions (A-B and B-C) demonstrating a learned intraverbal relation between value-congruent actions and their outcomes (A-B), as well as outcomes to participant values. Kenju and Ethan demonstrated the emergence of stated values to value-congruent actions (C-A) relations, while Diego required exemplar training for two C-A relations (C1-A1 and C2-A2) prior to achieving terminal mastery criteria.

From a derived intraverbal perspective, these results align with existing research demonstrating the emergence of untrained transitive intraverbal relations following a linear equivalence-based instruction training sequence (Conine et al., 2024), none of which have taught autistic individuals value-congruent intraverbals. Viewed through this lens, the present clinical evaluation offers a simple demonstration of how equivalence-based instruction can be used to promote derived intraverbal relations between client-identified values and corresponding value-congruent actions. A contrasting conceptualization is offered within the relational frame theory literature (Dixon et al., 2023; Paliliunas, 2022). However convenient to conceptualize the stimulus arrangement as one of equivalence, the relationship between stated value-congruent actions (A) and their stated outcomes (B) may be better conceptualized as temporal relations, where an action occurs that leads to the stated outcome (e.g., first I [action] then [outcome]). Further, the RFT conceptualization of values includes hierarchical relational responding (Paliliunas, 2022), where the derived relation between participant values (C) and spoken value-congruent action (A) may be conceptualized as one of hierarchy (i.e., action as *part of* or *belonging to* the value) rather than one of equivalence (i.e., functional substitutability).

Interestingly, Diego, who required exemplar training for C1-A1 and C2-A2 relations, scored only 50% (8 out of a possible 16) on the transformation expressive hierarchical framing subtest, while Kenju and Ethan scored 75% (12 out of 16) and 88% (14 out of 16), respectively. These data are consistent with a conceptualization of the relationship between values and expressive value-congruent action identification as one involving hierarchical framing as opposed to equivalence. Of course, this conceptualization relies on the RFT-based understanding of relational framing as a generalized relational operant (Dixon et al., 2021). Other variables may have resulted in differential outcomes for Diego relative to Kenju and Ethan, such as his high frequency of cancellations, changes in school placement, or another variable related to his co-occurring ADHD diagnosis. Future research should replicate the current protocol with additional participants exhibiting varying levels of hierarchical framing repertoires, specifically assessing the relationship between the establishment of hierarchical framing as a generalized relational operant and performance on tests for emergent relations between values and value-congruent action identification.

Regardless of theoretical interpretation, the practical utility of this teaching approach may aid in treatment protocols for clients more broadly. These findings may be useful for clinicians aiming to strengthen client repertoires for verbally identifying value-congruent actions aligned with their personal values, which is a skill central for effective comprehensive ACT interventions.

The current study is not without its limitations. First, it is possible that the outcomes may have differed for Diego using a one-to-many (OTM) or many-to-one (MTO) training structure, where participants might be trained on A-B and A-C relations with tests for emergent B-C and C-B relations (OTM), or on A-C and B-C relations with tests for emergent A-B and B-A relations (MTO), respectively. Although the present evaluation adhered to the linear training structure outlined in the PEAK E-10N program description, prior research has found that OTM and MTO training structures are more likely to produce positive equivalence outcomes (Ayres-Pereira & Arntzen, 2021).

Importantly, procedures used in this evaluation targeted only verbal relations (i.e., intraverbals) among value-congruent actions, their outcomes, and participant-identified values; the impact on actual value-congruent behavior was not assessed. Further, assessing generalizability of these findings would enhance their clinical relevance, as ACT aims to promote value-congruent behavior across a wide range of contexts. One potential avenue for future research is to examine client engagement in ACT exercises following relational training, as was the case in Gilsenan et al. (2022), where a relational training package targeting all six ACT core processes resulted in measurable increases in ACT engagement and reductions in value-incongruent behavior. Whether the current protocol produces similar effects related to values and committed action remains an empirical question. Future research is needed to determine whether this approach leads to meaningful changes in overt value-congruent actions. Finally, the inclusion of a systematic and technological procedure for values identification would strengthen extensions of the current analysis and may provide practitioners with the foundational skills necessary to support clients in identifying individualized values-congruent actions.

## Data Availability

All data reported in this study are stored in the Cognition, Behavior, and Mindfulness Lab at the University of Illinois, Chicago. These data are available from the second author upon request.
